# Nerve growth factor (NGF) with hypoxia response elements loaded by adeno-associated virus (AAV) combined with neural stem cells improve the spinal cord injury recovery

**DOI:** 10.1038/s41420-021-00701-y

**Published:** 2021-10-21

**Authors:** Qiuji Wu, Ziyue Xiang, Yibo Ying, Zhiyang Huang, Yurong Tu, Min Chen, Jiahui Ye, Haicheng Dou, Sunren Sheng, Xiaoyang Li, Weiyang Ying, Sipin Zhu

**Affiliations:** 1 Department of Orthopaedics, The Second Affiliated Hospital and Yuying Children’s Hospital of Wenzhou Medical University, Wenzhou, Zhejiang China; 2 Second Medical College of Wenzhou Medical University, Wenzhou, Zhejiang China; 3 Department of Pain Medicine, The Second Affiliated Hospital and Yuying Children’s Hospital of Wenzhou Medical University, Wenzhou, Zhejiang China

**Keywords:** Neural stem cells, Spinal cord injury

## Abstract

The ischemia and hypoxia microenvironment after spinal cord injury (SCI) makes SCI repair a challenging problem. With various stimulus, chances for neural stem cells (NSCs) to differentiate into neurons, astrocytes, oligodendrocytes are great and is considered as a potential source of the stem cell therapy to SCI. Our research used adeno-associated virus (AAV) to carry the target gene to transfect neural stem cells. Transfected NSCs can express nerve growth factor (NGF) navigated by five hypoxia-responsive elements (5HRE). Therefore, the 5HRE-NGF-NSCs could express NGF specifically in hypoxia sites to promote the tissue repair and function recovery. Based on the regeneration of neurocytes and promotion of the recovery found in SCI models, via locomotor assessment, histochemical staining and molecular examinations, our results demonstrated that 5HRE-NGF-NSCs could improve the motor function, neurons survival and molecules expression of SCI rats. Meanwhile, the downregulated expression of autophagy-related proteins indicated the inhibitive effect of 5HRE-NGF-NSCs on autophagy. Our research showed that 5HRE-NGF-NSCs contribute to SCI repair which might via inhibiting autophagy and improving the survival rate of neuronal cells. The new therapy also hampered the hyperplasia of neural glial scars and induced axon regeneration. These positive functions of 5HRE-NGF-NSCs all indicate a promising SCI treatment.

## Introduction

Spinal cord injury (SCI), an irreversible and devastating disease is a global health challenge for decades and lacks standard treatment [1–3]. The prolonged detriments caused by SCI contained two segments i.e., primary traumatic injury caused by direct damage and post-traumatic degradation provoked by secondary injury, including free radical formation, apoptosis, demyelination, autophagy, etc [4, 5]. Several events in secondary injury can cause neuronal death and microenvironment imbalance [6, 7]. Neuronal damage inevitably leads to a severe loss of sensory and motor functions, affecting patients’ quality of life [8, 9]. In short, a series of uncontrollable events exert an aggravated impact on SCI patients after primary injury.

Researches on SCI treatment mainly focus on neuron protection, axon regeneration, and angiogenesis [10–12]. Preceding in vivo researches indicated that neurons’ death and disruption are significant obstacles to recovery [13–15]. With breakthroughs in regeneration and restoration, stem cell transplantation is now recognized as a therapeutic option for SCI [16, 17]. The primary purpose of stem cell transplantation is to compensate for cell loss after SCI [18]. Neural stem cells (NSCs) could serve as an unlimited source for nerve cells generation [19, 20]. For example, NSCs can substitute for devitalized oligodendrocytes in the injured spinal cord [21]. Neural growth factor (NGF) is the first discovered factor, among the neurotrophin family [22] and serves as an indispensable part in the neural development of the central nervous system (CNS) and CNS conditions [23–25]. In vitro, NGF showed desirable neuroprotection and neuro-nutrition seemed relatively less in vivo [26]. However, giant weight and high-polarity of neurotrophins exhibit unfavorable biodistribution and inadequate bioavailability [27], therefore, it is less likely for neurotrophins to pass the blood-brain barrier (BBB), especially during systematic administration [28], and it becomes more unpredictable for delivery in CNS [29]. As five hypoxia-responsive elements (5HRE) help to release NGF in hypoxia position long-lasting and specifically, the adeno-associated virus (AAV) serves as a vector, providing a platform for nutrition support and controlled expression. In an ideal condition, we expected NSCs could also repair tissues defect. In order to enable NGF to repair effectively, we constructed 5HRE-NGF-NSCs. For the 5HRE-NGF-NSCs, we aimed to regulate the release of NGF by 5HRE, which helps to release NGF in the hypoxia positions (often the targeted positions). And the AAV serves as a vector, which can provide a platform for nutrition support and controlled expression. In this article, we omitted “AAV” when name groups because all the groups are based on the vector-based system. We also aimed to regulate the hypoxic microenvironment developed after injury and promote neuron survival and axon regeneration by regulating NGF expression by NSC through 5HRE [30, 31].

Apoptosis becomes normal in the chronic recovery phase but when injury continues [32], we turned our attention to autophagy. Autophagy is the degradation of the cytoplasm and induces nerve cell death in SCI [33]. We introduced LC3- II, Beclin and p62 to quantify the level of autophagy at the injury site. When autophagy is activated, modification of LC3 begins [34]. The process of LC3 formation starts with a precursor protein, proLC3. Then it is transformed into a proteolytically processed LC3-I structure LC3-II [35]. Accordingly, the activation of autophagy can be evaluated through the examination of LC3-II. Beclin 1 plays an essential role in autophagy. Its regulatory action on autophagy promotes Vps34, and its recruitment activity of membranes in autophagosomes formation [36, 37]. The p62, a substrate protein of the autophagic course, serves as a flux indicator of autophagy in vivo [38].

In this study, we explored a treatment for SCI that combines NGF with neural stem cells. Besides, we introduced the hypoxia-responsive elements to induce the transplanted NSC to express NGF direction. This study extends current knowledge of the stem cell research of SCI but is probably to be employed to confront the ischemic background that provokes excessive autophagy and impedes cell transplantation, and enhances the quality of spinal cord redevelopment.

## Results

### NSCs efficiently express NGF under the control of a hypoxia-inducible system

**Fig. 1:**
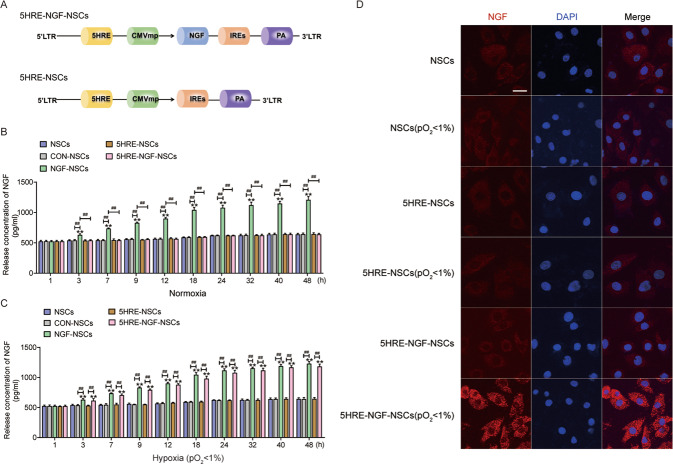
The establishment and authentication of adeno-associated virus-II loaded 5HRE-NSCs and 5HRE-NGF-NSCs. **A** A schematic plot of constructs of adeno-associated virus-II loaded 5HRE-NSCs and 5HRE-NGF-NSCs. **B**, **C** Release concentration of NGF under control and hypoxia(pO_2_ < 1%) conditions. Levels of NGF exhibited by ELISA. ** notes *P* < 0.01 versus NSCs group, ## notes *P* < 0.01 and # notes *P* < 0.5. Data are the mean values ± SD, *n* = 6. **D** Staining of NGF and nucleus, in NSCs, 5HRE-NSCs, 5HRE-NGF-NSCs and corresponding hypoxia (pO_2_ < 1%) groups, showing the NGF (red), nuclei (blue) in vitro. Scale bar = 50 μm.

We generated NSCs expressing NGF to regulate hypoxia using an AAV system (Fig. 1A). The release concentration of NGF in the NGF-NSCs group escalated steadily and distinctively within 48 h, under the normal condition, while the other three groups exhibited low levels like the NSCs groups. Meanwhile, under the hypoxia condition (pO_2_ < 1%), 5HRE-NGF-NSCs showed a high concentration like the NGF-NSCs group. Importantly, release in the 5HRE-NGF-NSCs group in controlled condition was much lower than under hypoxia condition, indicating that the hypoxia efficiently activated NGF expression with 5HRE gene (Fig. 1B, C). The immunofluorescence of NGF showed that there is no significant difference among NSCs, 5HRE-NSCs and their related hypoxia groups, however, 5HRE-NGF-NSCs (pO_2_ < 1%) showed higher expression of NGF than 5HRE-NGF-NSCs (Fig. 1D).

### The transplanted cells NGF expression detection

**Fig. 2:**
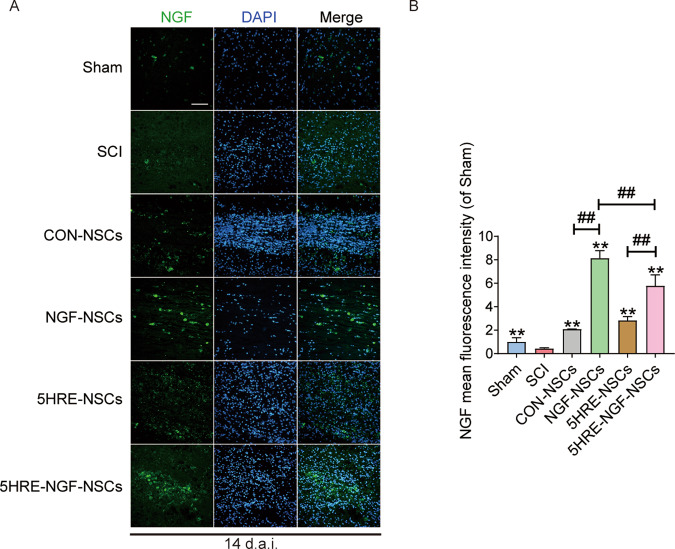
NGF-NSCs group and corresponding hypoxia regulated expressed the higher NGF than other groups, as the former was the highest at the day of 14. **A** NGF staining of the Sham, SCI, CON-NSCs, NGF-NSCs, 5HRE-NSCs, and 5HRE-NGF-NSCs group. The light green parts are NGF^+^ stained neurons, the nuclei are labeled by DAPI (blue). Magnification was ×20. **B** Analysis of positive cells in immunofluorescence staining. ** signify *P* < 0.01 versus the SCI group, ## signify *P* < 0.01. Data are the mean values ± SD, *n* = 6.

Immunofluorescent studies on NGF in the lesion zone in rats transplanted with NSCs were conducted at 14 days after SCI (Fig. 2). As shown in Fig. 2, NGF-NSCs and 5HRE-NGF-NSCs groups showed a higher expression of NGF, and it is noteworthy that these two groups compared to CON-NSCs and 5HRE-NSCs groups, could effectively express NGF in vivo. The results showed that: NSCs acted as the carrier of NGF gene therapy, delivering adequate NGF protein secretion to the surrounding area.

### AAV-5HRE-NGF-NSCs enhanced locomotor and hindlimb function recovery

**Fig. 3:**
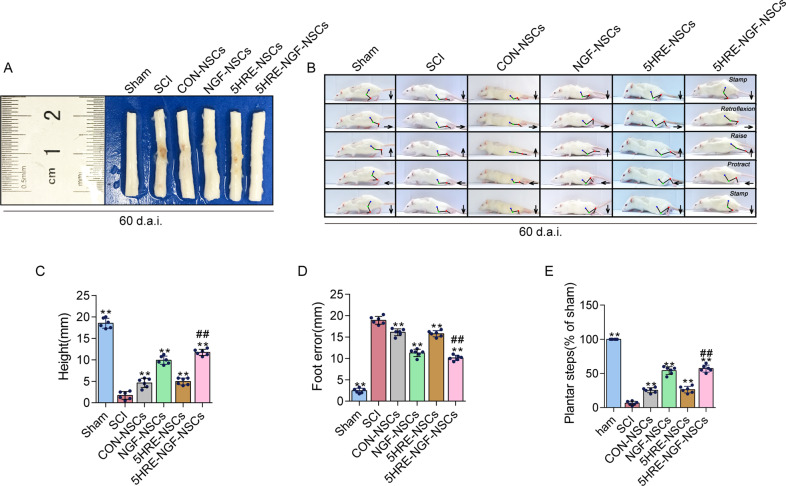
NGF promoted and protected the spinal cord tissues, improving the recovery of locomotor function. **A** Images of spinal cords in Sham, SCI, CON-NSCs, 5HRE-NSCs, 5HRE-NGF-NSCs group. **B** Video images sequence of rats 2 months after operation. Impaired hindlimb function while crawling is apparently manifested by powerless weight support (calculated as torso height minus ground), leg-extensor spasms (calculated as spasm interval relative to each step cycle duration), slow steps (quantity of hind-limb plantar steps each step cycle of forefoot) and weak foot placement (caudal to behind hip). Hip (iliac crest), knee and ankle joints are indicated by dots and lines. The arrow shows foot movement. Scale bar = 20 mm. **C–E** ** signifies *P* < 0.01 versus the SCI group, ## signifies *P* < 0.01 versus the NGF-NSCs group. Data are the mean values ± SD, *n* = 6.

**Fig. 4:**
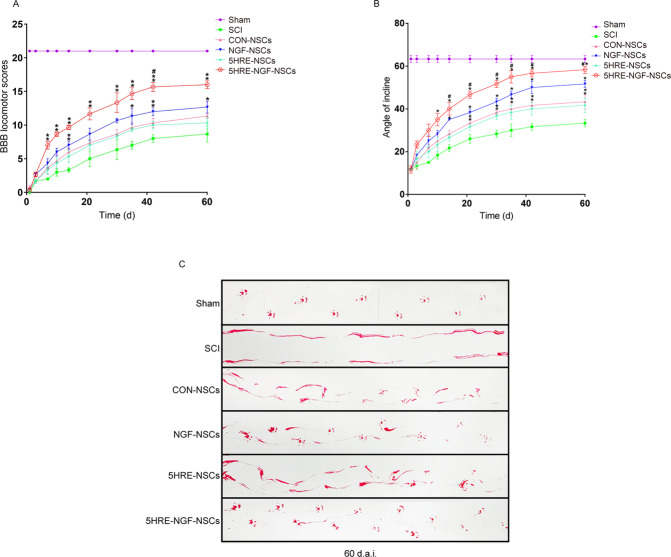
AAV-5HRE-NGF-NSCs enhanced the recovery after injury. **A** BBB rating scores of Sham, SCI, CON-NSCs, NGF-NSCs, 5HRE-NSCs, 5HRE-NGF-NSCs groups. The Sham group scores the highest point of 21, meaning a normal function. * is representative of *P* < 0.05 and ** of *P* < 0.01 versus the SCI group, # represents *P* < 0.05 versus the NGF-NSCs group. Data are the mean values ± SD, *n* = 6. **B** Inclined plane test scores of Sham, SCI, CON-NSCs, NGF-NSCs, 5HRE- NSCs, 5HRE-NGF-NSCs groups. * is representative of P < 0.05 and ** of *P* < 0.01 versus the SCI group, # represents *P* < 0.05 versus the NGF-NSCs group. Data are the mean values ± SD, *n* = 6. **C** Footprint analyses of Sham, SCI, CON-NSCs, NGF-NSCs, 5HRE -NSCs, 5HRE-NGF-NSCs groups. Sham group shows the normal movement. SCI group signifies the hind limbs dysfunction after SCI.

The tissue therapeutic effect of NGF transplantation on SCI was delivered through the anatomical appearance of injured spinal cords. Among four medication administered groups, the recovery of the injured spinal cord of NGF-NSCs and 5HRE-NGF-NSCs groups animals were better, while the repairing effect of 5HRE-NGF-NSCs was promising than NGF-NSCs (Fig. 3A). Using video image analysis to observe the rat’s crawling state and hind limb movement, we evaluated the parameters of height, foot error and plantar steps (Fig. 3B–E). The results showed that animals in the 5HRE-NGF-NSCs group had the best walking state. This result indicated that 5HRE-NGF-NSCs effectively improve animal motor function. Taking average scores of BBB and scores of inclined angles to assess locomotor ability, NGF-NSCs and 5HRE-NGF-NSCs groups vales were higher than other treated groups and between these two groups, 5HRE-NGF-NSCs was still the better (Fig. 4A, B). In the footprint test, rats in Sham group exhibited clear footstep traces which were natural and well-organized imprinting. Unlike the Sham group, there was no obvious footprint in the SCI group. Rats in four treated groups shown an improvement in their imprinting, animals in NGF-NSCs and 5HRE-NGF-NSCs groups were better than the other groups, as 5HRE-NGF-NSCs group rats showed a conspicuous rehabilitation in footprint (Fig. 4C).

**Fig. 5:**
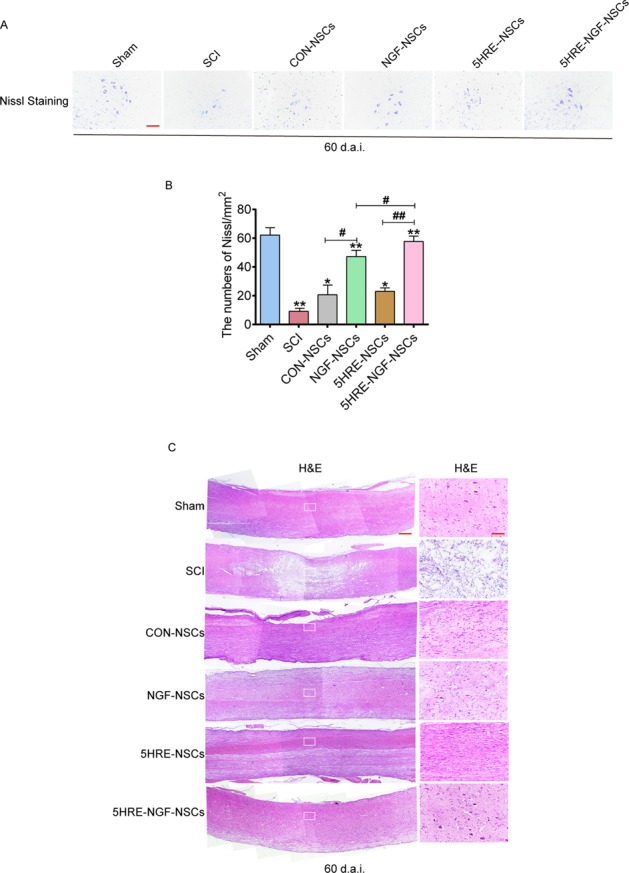
5HRE-NGF-NSCs achieved the maximum in improving the recovery of SCI. **A** Nissl staining results of transection spinal cord for the Sham, SCI, CON-NSCs, NGF-NSCs, 5HRE-GFP-NSCs, 5HRE-NGF-NSCs group, scale bar = 500 mm. **B** Analysis of H&E and Nissl staining of sections. * and ** signify *P* < 0.05 or *P* < 0.01 versus the Sham group, “#” and “##” signify *P* < 0.05 or *P* < 0.01 comparing between two groups. Data are the mean values ± SD, *n* = 6. **C** H&E and Nissl staining results of scored out spinal cord for the Sham, SCI, CON-NSCs, NGF-NSCs, 5HRE-NSCs, 5HRE-NGF-NSCs group, scale bar = 500 mm. Regions boxed manifests a representative region with higher power images, scale bar = 100 mm.

Histological analysis of longitudinal and coronal slices showed that four treatment methods could repair the spinal cord defect at various levels. The area of SCI was the smallest in 5HRE-NGF-NSCs samples (Fig. 5). In addition, the tissue structure of 5HRE-NGF-NSCs was clear and natural. In Nissl staining, amongst the four treated groups, the quantity of Nissl^+^ cells in the NGF-NSCs and 5HRE-NGF-NSCs groups was higher; therein, NGF-5HRE-NSCs was the better. These results indicated that 5HRE-NGF-NSCs more effectively repaired the injured spinal cord tissue and increased neurons.

### AAV-5HRE-NGF-NSCs improved the expression of NeuN protein-positive neurons in SCI rats

**Fig. 6:**
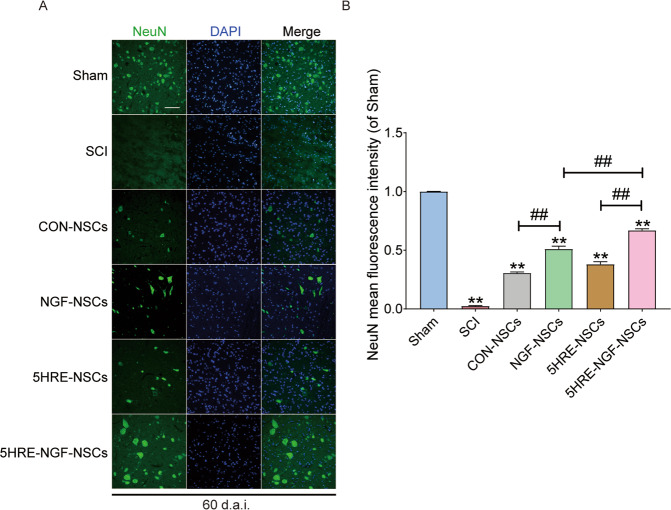
Implement of NSCs and NGF, and application of hypoxia condition increased the expression of NeuN^+^ proteins at the day of 60. **A** Immunofluorescence staining of NeuN in spinal cord lesions. The nuclei are labeled by DAPI (blue). The bright green dots are NeuN^+^ proteins, magnification was ×20. **B** Analysis of positive cells in immunofluorescence staining. ** signify *P* < 0.01 versus the Sham group, ## signify *P* < 0.01. Data are the mean values ± SD, *n* = 6.

Using immunofluorescent staining for NeuN proteins visualizing neuronal nucleus, we found that after injury, NeuN^+^ signals in SCI was plunged while much more positive signals were observed in 5HRE-NGF-NSCs samples (Fig. 6). The above results indicated that 5HRE-NGF-NSCs could up-regulate the NeuN proteins, suggesting the neuroprotective and neuron modulation functions of 5HRE-NGF-NSCs.

### AAV-5HRE-NGF-NSCs impeded the autophagic cell death course after SCI

**Fig. 7:**
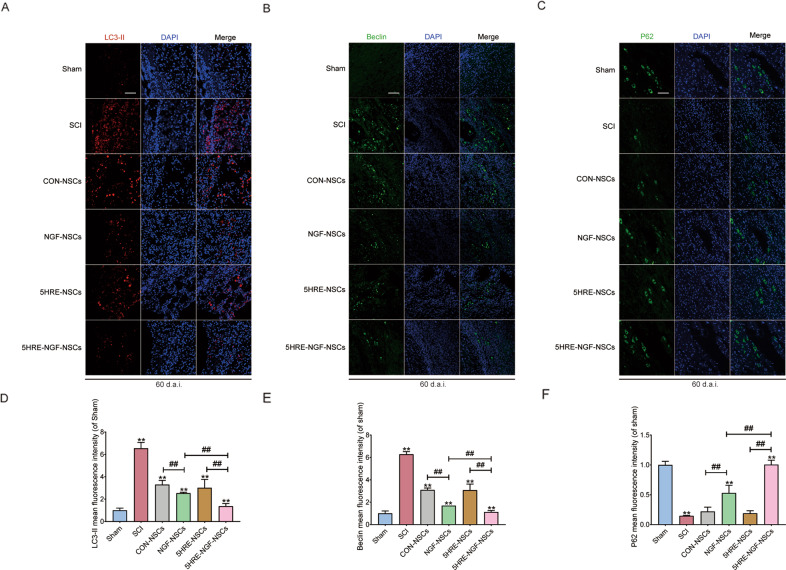
The application of NSCs, NGF, and the hypoxia inhibited autophagic cell death in SCI animals. **A–C** LC3-II, Beclin, P62 staining of the Sham, SCI, CON-NSCs, CON-NGF-NSCs, 5HRE-NSCs, and 5HRE-NGF-NSCs group. The red dots are LC3-II^+^ stained neurons, green dots in **B** signifies the Beclin^+^ stained neurons and in **C** signifies the P62. The nuclei demonstrating by DAPI (blue). Magnification was ×20. **D–F** Analysis of positive cells in immunofluorescence staining. ** signify *P* < 0.01 versus the Sham group, ## signify *P* < 0.01. Data are the mean values ± SD, *n* = 6.

The autophagy signal pathway proteins (LC3-II, Beclin, P62) was detected and analyzed at day 60. In previous studies, LC3-II as an autophagy-related protein was applied, which is of great importance in the autophagy process [39, 40]. Comparing the chronic recovery in the Sham and SCI groups, the process of excessive autophagy was stimulated after SCI (Fig. 7A, D), which aggravated the damage caused by SCI and compromise the regeneration. Beclin was confirmed as one of the proteins promoting excessive autophagy [41], combining various regulatory factors that induced autophagy [36]. P62 is a protein located in nuclei, which combines with intracellular ubiquitin [42], then the LC3-II and eventually digested in the lysosome. Unlike the other two proteins, P62 is negatively correlated with autophagy [43], which plunged after injury. As grafted the NSCs, the LC3-II^+^ (Fig. 7A, D) and Beclin (Fig. 7B, E) signals in four treated groups decreased, while P62 increased, and 5HRE-NGF-NSCs group exhibited more intensive inhibitory action than 5HRE-NSCs group on autophagy, indicating the excessive NGF enhanced the recovery. We also found that positive signals were greater in 5HRE-NGF-NSCs samples than in NGF-NSCs, indicating that NGF is accurately released to hypoxia sites and inhibit excessive autophagy.

## Discussion

Recently, stem cell therapy has been a perspective option for nervous system diseases. After SCI, neuron losses, and given the non-regenerative property, replenishing exogenous neurons can be a potential strategy to treat SCI. Among all stem cells, neuronal stem cells have outstanding multipotent differentiative properties [44–47]. Previous studies have reported that stem cells could replenish exogenous neurons through transplantation and compensate for lost neurons [18, 48]. In this study, we transplanted NSC into the injured site and improved neuron quantity in CON-NSCs and 5HRE-NSCs groups rats, but we cannot find the ideal number of neurons (Fig. 6B). Meanwhile, rats in NGF-NSCs and 5HRE-NGF-NSCs groups showed an increased number of neurons (Fig. 5B) and a higher level of NGF (Fig. 2B) than CON-NSCs and 5HRE-NSCs groups.

In conclusion, the overexpressed NGF may account for the disparity between these two parties. In the current study, the overexpressed NGF by 5HRE-NGF-NSCs and by NGF-NSCs provided stable NGF sources. There are reasonable grounds for us to believe that the exogenous NGF helps NSCs improve SCI recovery. Researches have stated that, as one of neurotrophins, NGF increase neurotrophic support and neuronal protective effects [49, 50]. Moreover, the desirable locomotor recovery of SCI animals was observed after NGF administration, suggesting that NGF improved the recovery of SCI (Figs. 3B-E and 4). Nowadays, with viral vector systems, gene therapy becomes more sustained and stable. Moreover, based on viral vectors, therapeutic genes can be efficiently overexpressed, achieving more ideal recovery outcomes. However, without high specific regulators, the overexpression NGF could be unpredictable and nonselective. Many researches have reported that overexpressed NGF is latently oncogenic and possesses other risks [51, 52], and thus needed appropriate regulators to improve the specificity of NGF overexpression. HRE is induced explicitly by hypoxia-inducible factors in hypoxia sites [53]; thereupon, according to injury conditions, we hope to regulate the expression of NGF so that an appropriate amount of NGF could be expressed explicitly at hypoxia sites and prevent latent hyperplasia or other side-effects.

We constructed the AAV system and found that 5HRE-NGF-NSCs group have better locomotor recovery than the NGF-NSCs group, indicating that, under HRE regulation, NGF could provide a better therapeutic effect. In the current study, the graft of 5HRE-NGF-NSCs specifically affected the hypoxic loci, surprisingly decreased neurons injury and loss, and promoted the recovery of dynamic functions. Furthermore, the release of NGF was enhanced by hypoxia condition and normal condition showed no distinct difference, indicating that the 5HRE regulation is of highly accurate. Moreover, under the regulation of HRE, a more suitable concentration could be provided, and as the progress of recovery process, the release of NGF could adapt to the actual condition. Thus, the resilient amount of NGF regulated by HRE can prevent the possible glioma provoked by unlimited NGF release [54, 55].

We observed the repair potential of NGF, and we are curious to further explore the exact mechanisms of NGF. Previously, proteins associated with apoptosis and autophagy are both observed in the acute stage of SCI recovery [56, 57]. However, during the chronic SCI recovery process, apoptosis, an irritable activity, gradually decrease [32], but injury continues. Thus, we turned our attention to autophagy. Autophagy is a cell homeostasis regulator, which exists for the long term [58]. It is stated that autophagy was overstimulated during the chronic recovery stage and then resulted in a high cell death rate [59, 60]. In our study, we found that Beclin ascended after SCI, and its downstream molecule LC3-II was also up-regulated, and the substrate of LC3-II (P62) [61] was down-regulated. Beclin has been reported to be involved in autophagy process [62]. When autophagy is stimulated, Beclin overexpressed and reacts to its upstream molecules [41], provoking the overstimulated autophagy in the chronic recovery stage. Beclin serves as a key target to inhibit excessive autophagy after SCI. We found that NGF-NSCs and 5HRE-NGF-NSCs effectively inhibited the release of Beclin (Fig. 7B). We further detected the expression of LC3-II and P62; the former rose after injury, while the latter plunged. After the administration of 5HRE-NGF-NSCs, LC3-II decreased and P62 increased. In short, 5HRE-NGF-NSCs has a regulatory effect on autophagy.

There are different evidences and arguments about the role of autophagy in practical therapies [46, 63–65]. Thus, its role is still controversial. Our results indicated that inhibiting autophagy in the chronic recovery stage could improve the recovery of SCI. In the microenvironment after SCI, with adverse factors, autophagy is likely to happen, which may deepen the damage and cause irreversible defects as evident by histology and functions of sensory and locomotor. In general, the current study results on autophagy is not profound enough; therefore, we aimed to explore the relationship among autophagy, NGF and NSC in our future study.

Our research firstly transplanted 5HRE-NGF-NSCs into the SCI animals and found that this system improves the recovery of SCI models, which signified the high possibility of treatment using adenovirus vector and NGF, NSCs under the control of hypoxia-responsive elements. Therefore, a complete modifying and regulating system for NGF and NSCs is of great importance. How to turn adverse environment like hypoxia into “support platform” or functioning factor may be a promising method for future study.

## Materials and methods

### Reagents and antibodies

NSCs culture medium was under support from Chi Scientific (Jiangsu, China). Anti-NGF, anti-LC3-II, anti-Beclin1, anti-P62, anti-NeuN, donkey anti-rabbit IgG and anti-mouse IgG with Alexa Fluor 594 (red) and Alexa-Fluor 488 (green) were supplied by Abcam (Cambridge, Britain). DAPI (4′,6-diamidino-2-phenylindole), a fluorescent agent used for nuclear staining, was purchased from Sigma-Aldrich (St. Louis, MO, USA).

### Insulation and culture of rat embryonic derived NSCs

Acquisition of Sprague Dawley rat embryos (14–16 days after pregnancy) was under the conventional surgical procedure, which were afterwards followed by isolation and the immersion of cerebral cortex of each embryo in D-Hanks solution. Under a microscope, remove the cerebrovascular and meninges, and centrifuge at 1000 rpm (3 min). Next, the cerebral cortex was rinsed and treated with trypsin (0.125%) and EDTA (0,0102%). Using culture medium DMEM containing 1% N2 supplements, 2% B27, 20 ng/ml bFGF, 20 ng/mL EGF, 200 IU/L penicillin and 100 IU/L streptomycin, the trypsinization was aborted, and then cell suspension was collected and centrifuged. After finishing the re-suspension of cell culture with medium, cells were shifted to NSC suspension culture medium at 37˚C, in humid condition containing 5% CO_2_, and the culture was continued.

### Construction of recombinant adeno-associated viral vector containing NGF

Out sourcing service supplied AAV virus particles. Briefly, AAV-5HRE-NGF and AAV-5HRE plasmids, together with pAAV-RC and the helper plasmid, were transfected and produced in HEK293 cells. The virus particles were purified by cesium chloride density gradient followed by dialysis. Next, a quantitative polymerase chain reaction (qPCR) was performed later to quantify the titers of the virus. The results indicated that AAV, AAN-NGF, AAV-5HRE, and AAV-5HRE-NGF contained virus particles of 2.30 × 10^12^, 2.27 × 10^12^, 2.66 × 10^12^, 2.50 × 10^12^, and 2.45 × 10^12^/ml, respectively. Then, NSCs were added in both groups to transduce under 1 × 10^5^ multiplicity of infection (MOI), which was repeated until the transduction rate reached 90%. Transduced cells were cultured in normoxia and hypoxia (<1% O_2_) environments to realize hypoxia-induced expression for no less than 6 h. Hypoxia conditioning was performed in a hypoxic incubator for 6 h and then hypoxia groups were incubated under normoxic condition for 42 h. The protein concentration of cultured supernatant was quantified using the NGF enzyme-linked immunosorbent assay (ELISA) kit (BeyoLife, Shanghai, China).

### Adeno-associated virion transduction

NSCs were inoculated in 24-well plates (2 × 10^5^ per well), which then were under exposure of AAV, AAV-NGF, AAV-5HRE, and AAV-5HRE-NGF (in DMEM), at 37 °C inoculums for 24 h. After removing the inoculum, cells were rinsed with DMEM once, which eliminated transduce-failed cells. The expression of NGF was verified using immunofluorescence staining, confocal analyses and ELISA.

### ELISA

Supernatants were collected and performed ELISA to ensure the NGF concentrate. Within 48 h after damage, we obtained NSCs at an array of time points (1, 3, 7, 9, 12, 18, 24, 32, 40, 48 h) for ELISA. NSCs were centrifuged at 12,000 rpm for 10 min and then acquired the supernatants for ELISA. The density of protein in supernatants or tissue homogenates were evaluated by Micro BCA Protein Assay kits (Pierce, Rockford, IL); moreover, the counterpart was supplemented on a 96-well plate (coated with designative antibodies). The analysis of NGF density was consistent with the instruction of NGF ELISA kits (BeyoLife, Shanghai, China).

### Animal model of spinal cord injury

Seventy-two (HE staining and Nissl staining require 4 SD rats in each group, and immunofluorescence staining requires 6 SD rats in each group. In addition to the loss due to rat death during the experiment, 12 SD rats in each group are required. Hemiplegia and dead rats excluded from group.) adult female SD rats (220–250 g) were supplied by Animal Center of the Chinese Academy of Sciences, Shanghai, China. With the sanction of the Animal Care and Use Committee of Wenzhou Medical University, all the animal experiments complied with the National Institutes of Health guidelines. Animals were randomly assigned in equal amounts to six groups, comprising Sham, SCI, CON-NSCs, NGF-NSCs, 5HRE-NSCs, and 5HRE-NGF-NSCs. Formerly, animals were housed for 7 days, catering with suitable water and food, in an alternative light and shaded environment per 12 h. The animals were anaesthetized with 5% isoflurane until unconscious. Implemented the incision on the skin along the midline of the back and the resection of the 8–10th thoracic spinal vertebrae, except the Sham group, additional operation on other five groups rats were a free-fall strike on T9 segment of the spinal cord with a 10 g hammer from the height of 25 mm. The postoperative care contained discharging of bladders twice per day (fixed time in morning and evening) and twice injections of the cefazolin sodium (50 mg/kg, i.p.). Rats were placed at plastic cages equipped with a regular moderate diet.

### Transplantation

Transplantation was conducted on the 7^th^ day after injury when the inflammatory reaction was subtle and the glial scar was in the initial stage. The inflammation broke the balance of the microenvironment and impeded grafted cells’ survival; meanwhile, the glial scar blocked the communication between transplant and original tissue. The animal was fixed with a rat vertebra-holder (Cunningham spinal adaptor, Stoelting Co., Wood Dale, IL), and the T9 segment of spinal cord was exposed. 2 × 10^5^ NSCs cells/10 μl were transfused in the position, using 25 μl microinjector (26 G, an inner diameter of 0.24 mm, an outer diameter of 0.6 mm, 30° bevel, 1 cm long needle). The procedure of cells acquisition was: a Nano-Injector (Stoelting Co.) at a speed of insert is 1 μL/min and at a depth of 1 mm into the epicenter below the dorsal surface was inserted to acquire cells, which was then incubated in inoculum suspension for transplantation preparation. The quantity of cells was determined, referring to our initial research that comprised 2 × 10^5^ NSCs cells/10 μl transfused into the proximal, central, and distal parts of the injured spinal cord. After injection, the microinjector was positioned for 5 min, in case of the suspension leak. Moreover, the control group was treated with 10 μl phosphate buffered saline (PBS). Moreover, cyclosporine (10 mg/kg dose) that serves as immunosuppression were intraperitoneally injected daily until the day 60.

### Functional recovery evaluation of SCI rats

To understand the therapeutic effect, animals’ spinal cord nerve function in all groups was quantified by BBB score (at day 1, 3, 7, 10, 14, 21, 30, 35, 42, 60), the oblique plate test (at day 60), footprint (at day 60) and video recording images (at day 60). For BBB scoring, 3 independent inspectors blinded the experimental conditions and obtained the measurement results. The approaches were detailed: the rats were first set on a test bench that enabled them to crawl without restriction, and their limb walking action was recorded, especially the hindlimb action. The inclined plate test was conducted as follows: one inclined plate with adjusted structure was set on a desk; Place the rats on a slide-proof pad (thickness: 6 mm), and the longitudinal axis of rats was kept at a right angle to the long axis of the inclined plate; Gradually elevated the inclined plate until the plate with the tilt angle can support rats for 5 sec and obtained the results. A track (7.5 cm × 100 cm) was preset and the pedestal surface was overlaid with white paper. To avoid the light and to establish a dark environment where rats adapt, black plastic film was mantle over the track. The red dye was used to visualize rats’ hind footprint. Begin at one end of the track, assuring the animals to complete the entire route. The footprint could reflect the locomotor recovery. The images from video and quantitative analysis were followed that include weight support, leg extensor spasms, footsteps quantity, as well as the posture of the foot.

### Hematoxylin and eosin staining and Nissl staining

On day 60 (after the surgery), 5% isoflurane was used to anaesthetize rats. The heart was perfused with 0.9% NaCl and then injected with the 500 ml 4% paraformaldehyde solution. A level resection was performed at the site of 8–10th thoracic spinal vertebral where contained the stroke position. The segments excised were marinated in 4% paraformaldehyde for 24 h and then embedded in paraffin. For further histopathological dimension examination, transverse paraffin sections (10 μm) were colored with hematoxylin and eosin (H&E). Meanwhile, for Nissl staining, slices were positioned in the incubator with 1% cresyl violet. Under an optical microscope, the above two types of sections were observed and scan.

### Immunofluorescence staining

In an incubator containing bovine serum albumin (BSA) and 0.1% Triton X-100 attenuated with PBS at 37 °C, sections were incubated for 1 h and then incubated with aiming primary antibodies in the same condition at 4 °C overnight. We used anti-NeuN (1:500), anti-NGF (1:500), anti-LC3-II (1:500), anti-P62 (1:500), and anti-Beclin (1:1000) as primary antibodies to track neurocytes in separate experiments. In subsequent staining, firstly washed with PBS three times (per 5 min), then sections were incubated in incubators containing secondary antibodies under a dark environment for 1 h. After duplicate staining, nuclei were dyed with DAPI (0.25 μg/ml). Images were captured by Nikon ECLIPSE Ti microscope (Nikon, Tokyo, Japan).

### Statistical analysis

The data were presented as mean ± standard error mean (SD). In the analysis of two drug treatment groups, a Student’s t-test was used to determine differences, with a significant value of *P* < 0.05. If treatment groups are more than two, one-way analysis of variance (ANOVA) and Dunnett’s post hoc test was used to quantify data, with a significant value of *P* < 0.05.

## Supplementary information

Editorial Certificate

## Data Availability

The data used to support the findings of this study are available from the corresponding author upon request.

## References

[1] The global, regional, and national burden of colorectal cancer and its attributable risk factors in 195 countries and territories, 1990–2017: a systematic analysis for the Global Burden of Disease Study 2017. The lancet. Gastroenterology & hepatology 4, 913–33 (2019). 10.1016/S2468-1253(19)30345-0 PMC7026697 31648977

[2] Lee B, Cripps R, Fitzharris M, Wing P (2014). The global map for traumatic spinal cord injury epidemiology: update 2011, global incidence rate. Spinal cord..

[3] Feigin, V, Vos, T, Alahdab, F, Amit, A, Bärnighausen, T, Beghi, E et al. Burden of neurological disorders across the US from 1990–2017: A Global Burden of Disease Study. JAMA neurology (2020). 10.1001/jamaneurol.2020.4152 PMC7607495 33136137

[4] Silva N, Sousa N, Reis R, Salgado A (2014). From basics to clinical: a comprehensive review on spinal cord injury. Prog. Neurobiol..

[5] Ahuja C, Wilson J, Nori S, Kotter M, Druschel C, Curt A (2017). Traumatic spinal cord injury. Nat. Rev. Dis. Prim..

[6] Hernandez-Gerez E, Fleming I, Parson S (2019). A role for spinal cord hypoxia in neurodegeneration. Cell death Dis..

[7] Beattie M (2004). Inflammation and apoptosis: linked therapeutic targets in spinal cord injury. Trends Mol. Med..

[8] Mohammed H, Hollis E (2018). Cortical reorganization of sensorimotor systems and the role of intracortical circuits after spinal cord injury. NeuroTherapeutics.

[9] Spinal Cord Injury (SCI) 2016 Facts and Figures at a Glance. The journal of spinal cord medicine 39, 493-4 (2016). 10.1080/10790268.2016.1210925 PMC5102286 27471859

[10] Amo-Aparicio J, Sanchez-Fernandez A, Li S, Eisenmesser E, Garlanda C, Dinarello C (2021). Extracellular and nuclear roles of IL-37 after spinal cord injury. Brain, Behav., Immun..

[11] Tsata, V, Möllmert, S, Schweitzer, C, Kolb, J, Möckel, C, Böhm, B et al. A switch in pdgfrb cell-derived ECM composition prevents inhibitory scarring and promotes axon regeneration in the zebrafish spinal cord. Developmental cell (2020). 10.1016/j.devcel.2020.12.009 33412105

[12] Zhou T, Zheng Y, Sun L, Badea S, Jin Y, Liu Y (2019). Microvascular endothelial cells engulf myelin debris and promote macrophage recruitment and fibrosis after neural injury. Nat. Neurosci..

[13] Liu S, Sarkar C, Dinizo M, Faden A, Koh E, Lipinski M (2015). Disrupted autophagy after spinal cord injury is associated with ER stress and neuronal cell death. Cell death Dis..

[14] Hutson T, Di Giovanni S (2019). The translational landscape in spinal cord injury: focus on neuroplasticity and regeneration. Nat. Rev. Neurol..

[15] Tran A, Warren P, Silver J (2018). The biology of regeneration failure and success after spinal cord injury. Physiological Rev..

[16] Vismara I, Papa S, Rossi F, Forloni G, Veglianese P (2017). Current options for cell therapy in spinal cord injury. Trends Mol. Med..

[17] Chhabra H, Sarda K (2017). Clinical translation of stem cell based interventions for spinal cord injury - Are we there yet?. Adv. Drug Deliv. Rev..

[18] Sahni V, Kessler J (2010). Stem cell therapies for spinal cord injury. Nat. Rev. Neurol..

[19] Pluchino S, Smith J, Peruzzotti-Jametti L (2020). Promises and limitations of neural stem cell therapies for progressive multiple sclerosis. Trends Mol. Med..

[20] Carradori D, Eyer J, Saulnier P, Préat V, des Rieux A (2017). The therapeutic contribution of nanomedicine to treat neurodegenerative diseases via neural stem cell differentiation. Biomaterials.

[21] Sontag C, Uchida N, Cummings B, Anderson A (2014). Injury to the spinal cord niche alters the engraftment dynamics of human neural stem cells. Stem Cell Rep..

[22] LEVI-MONTALCINI R, HAMBURGER V (1951). Selective growth stimulating effects of mouse sarcoma on the sensory and sympathetic nervous system of the chick embryo. J. Exp. Zool..

[23] Rafii M, Tuszynski M, Thomas R, Barba D, Brewer J, Rissman R (2018). Adeno-associated viral vector (Serotype 2)-nerve growth factor for patients with Alzheimer disease: a randomized clinical trial. JAMA Neurol..

[24] Kim M, Vargas M, Harlan B, Killoy K, Ball L, Comte-Walters S (2018). Nitration and glycation turn mature NGF into a toxic factor for motor neurons: a role for p75 and RAGE signaling in ALS. Antioxid. Redox Signal..

[25] Feczkó T, Piiper A, Ansar S, Blixt F, Ashtikar M, Schiffmann S (2019). Stimulating brain recovery after stroke using theranostic albumin nanocarriers loaded with nerve growth factor in combination therapy. J. Controlled Release.: Off. J. Controlled Release. Soc..

[26] Xu, D, Wu, D, Qin, M, Nih, L, Liu, C, Cao, Z et al. Efficient delivery of nerve growth factors to the central nervous system for neural regeneration. Advanced materials (Deerfield Beach, Fla.) 31, e1900727 (2019). 10.1002/adma.201900727 31125138

[27] Faustino C, Rijo P, Reis C (2017). Nanotechnological strategies for nerve growth factor delivery: therapeutic implications in Alzheimer’s disease. Pharmacol. Res..

[28] Kuo Y, Lee Y (2016). Rescuing cholinergic neurons from apoptotic degeneration by targeting of serotonin modulator-and apolipoprotein E-conjugated liposomes to the hippocampus. Int. J. Nanomed..

[29] Pardridge W (2015). Targeted delivery of protein and gene medicines through the blood-brain barrier. Clin. Pharmacol. Therapeutics.

[30] Rhim T, Lee D, Lee M (2013). Hypoxia as a target for tissue specific gene therapy. J. Controlled Release.: Off. J. Controlled Release. Soc..

[31] Zhu S, Chen M, Deng L, Zhang J, Ni W, Wang X (2020). The repair and autophagy mechanisms of hypoxia-regulated bFGF-modified primary embryonic neural stem cells in spinal cord injury. Stem Cells Transl. Med..

[32] Chen Z, Fu Q, Shen B, Huang X, Wang K, He P (2014). Enhanced p62 expression triggers concomitant autophagy and apoptosis in a rat chronic spinal cord compression model. Mol. Med. Rep..

[33] Gonzalez Porras M, Sieck G, Mantilla C (2018). Impaired autophagy in motor neurons: a final common mechanism of injury and death. Physiol. (Bethesda, Md.).

[34] Matsuzawa-Ishimoto Y, Hwang S, Cadwell K (2018). Autophagy and Inflammation. Annu. Rev. Immunol..

[35] Abbaszadeh F, Fakhri S, Khan H (2020). Targeting apoptosis and autophagy following spinal cord injury: Therapeutic approaches to polyphenols and candidate phytochemicals. Pharmacol. Res..

[36] Hill S, Wrobel L, Rubinsztein D (2019). Post-translational modifications of Beclin 1 provide multiple strategies for autophagy regulation. Cell Death Differ..

[37] Antonioli M, Di Rienzo M, Piacentini M, Fimia GM (2017). Emerging mechanisms in initiating and terminating autophagy. Trends Biochem. Sci..

[38] Zhou, K, Sansur, C, Xu, H & Jia, X. The temporal pattern, flux, and function of autophagy in spinal cord injury. *Int. J. Mol. Sci.* 18 (2017). 10.3390/ijms18020466 PMC5343998 28230791

[39] Lee Y, Lee J (2016). Role of the mammalian ATG8/LC3 family in autophagy: differential and compensatory roles in the spatiotemporal regulation of autophagy. BMB Rep..

[40] Sora V, Kumar M, Maiani E, Lambrughi M, Tiberti M, Papaleo E (2020). Structure and dynamics in the ATG8 family from experimental to computational techniques. Front. Cell Dev. Biol..

[41] Sun Y, Yao X, Zhang Q, Zhu M, Liu Z, Ci B (2018). Beclin-1-dependent autophagy protects the heart during sepsis. Circulation.

[42] Lamark T, Svenning S, Johansen T (2017). Regulation of selective autophagy: the p62/SQSTM1 paradigm. Essays Biochem..

[43] Komatsu M, Kageyama S, Ichimura Y (2012). p62/SQSTM1/A170: physiology and pathology. Pharmacol. Res..

[44] Li X, Dai J (2018). Bridging the gap with functional collagen scaffolds: tuning endogenous neural stem cells for severe spinal cord injury repair. Biomater. Sci..

[45] Yokota K, Kobayakawa K, Kubota K, Miyawaki A, Okano H, Ohkawa Y (2015). Engrafted neural stem/progenitor cells promote functional recovery through synapse reorganization with spared host neurons after spinal cord injury. Stem Cell Rep..

[46] Rong Y, Liu W, Wang J, Fan J, Luo Y, Li L (2019). Neural stem cell-derived small extracellular vesicles attenuate apoptosis and neuroinflammation after traumatic spinal cord injury by activating autophagy. Cell Death Dis..

[47] Lu P, Kadoya K, Tuszynski M (2014). Axonal growth and connectivity from neural stem cell grafts in models of spinal cord injury. Curr. Opin. Neurobiol..

[48] Mothe A, Tator C (2012). Advances in stem cell therapy for spinal cord injury. J. Clin. Investig..

[49] Keefe, K, Sheikh, I & Smith, G. Targeting neurotrophins to specific populations of neurons: NGF, BDNF, and NT-3 and their relevance for treatment of spinal cord injury. *Int. J. Mol. Sci*. 18 (2017). 10.3390/ijms18030548 PMC5372564 28273811

[50] Chen Z, Wang H, Yuan F, Zhang X, Dong X, Xie R (2016). Releasing of herpes simplex virus carrying NGF in subarachnoid space promotes the functional repair in spinal cord injured rats. Curr. gene Ther..

[51] Tzeng, H, Lin, S, Thadevoos, L, Ko, C, Liu, J, Huang, Y et al. The mir-423-5p/MMP-2 axis regulates the nerve growth factor-induced promotion of chondrosarcoma metastasis. Cancers 13 (2021). 10.3390/cancers13133347 PMC8268073 34283074

[52] Lin, H, Huang, H, Yu, Y, Chen, W, Zhang, S & Zhang, Y. Nerve growth factor regulates liver cancer cell polarity and motility. Mol. Med. Reports 23 (2021). 10.3892/mmr.2021.11927 PMC7905331 33649819

[53] Dou B, Zheng X, Tan D, Yin X (2020). The effect of HRE-regulated VEGF expression and transfection on neural stem cells in rats. Front. Cell Dev. Biol..

[54] Reichardt L (2006). Neurotrophin-regulated signalling pathways. Philos. Trans. R. Soc. Lond. Ser. B, Biol. Sci..

[55] Lin W, Liang W, Lee Y, Chuang S, Tseng T (2010). Antitumor progression potential of caffeic acid phenethyl ester involving p75(NTR) in C6 glioma cells. Chem.-Biol. Interact..

[56] Nicola F, Marques M, Odorcyk F, Arcego D, Petenuzzo L, Aristimunha D (2017). Neuroprotector effect of stem cells from human exfoliated deciduous teeth transplanted after traumatic spinal cord injury involves inhibition of early neuronal apoptosis. Brain Res..

[57] He Z, Zhou Y, Huang Y, Wang Q, Zheng B, Zhang H (2017). Dl-3-n-butylphthalide improves functional recovery in rats with spinal cord injury by inhibiting endoplasmic reticulum stress-induced apoptosis. Am. J. Transl. Res..

[58] Murrow L, Debnath J (2013). Autophagy as a stress-response and quality-control mechanism: implications for cell injury and human disease. Annu. Rev. Pathol..

[59] Lipinski M, Wu J, Faden A, Sarkar C (2015). Function and mechanisms of autophagy in brain and spinal cord trauma. Antioxid. Redox Signal..

[60] Hao H, Wang L, Guo Z, Bai L, Zhang R, Shuang W (2013). Valproic acid reduces autophagy and promotes functional recovery after spinal cord injury in rats. Neurosci. Bull..

[61] Narendra D, Kane L, Hauser D, Fearnley I, Youle R (2010). p62/SQSTM1 is required for Parkin-induced mitochondrial clustering but not mitophagy; VDAC1 is dispensable for both. Autophagy.

[62] Kanno H, Ozawa H, Sekiguchi A, Itoi E (2009). Spinal cord injury induces upregulation of Beclin 1 and promotes autophagic cell death. Neurobiol. Dis..

[63] He M, Ding Y, Chu C, Tang J, Xiao Q, Luo Z (2016). Autophagy induction stabilizes microtubules and promotes axon regeneration after spinal cord injury. Proc. Natl Acad. Sci. USA.

[64] Saraswat Ohri S, Bankston A, Mullins S, Liu Y, Andres K, Beare J (2018). Blocking autophagy in oligodendrocytes limits functional recovery after spinal cord injury. J. Neurosci.: Off. J. Soc. Neurosci..

[65] Wang J, Rong Y, Ji C, Lv C, Jiang D, Ge X (2020). MicroRNA-421-3p-abundant small extracellular vesicles derived from M2 bone marrow-derived macrophages attenuate apoptosis and promote motor function recovery via inhibition of mTOR in spinal cord injury. J. Nanobiotechnology.

