# Supplementary material for: Nerve growth factor (NGF) with hypoxia response elements loaded by adeno-associated virus (AAV) combined with neural stem cells improve the spinal cord injury recovery
Source: Cell Death Discov. 2021 Oct 21;7:301. doi: 10.1038/s41420-021-00701-y (PMC8531363; doi:10.1038/s41420-021-00701-y)
Supplement: Supplementary file 1 — Editorial Certificate [file 41420_2021_701_MOESM1_ESM.pdf]

AJESCI

EDITORIAL CERTIFICATE

This document certifies that the manuscript listed below was edited for proper English language, grammar, punctuation, spelling, and overall style by one or more of the highly qualified native English speaking editors at AJESCI.

Manuscript

Nerve growth factor (NGF) with hypoxia response elements loaded by adeno-associated virus (AAV) combined with neural stem cells improve the spinal cord injury recovery

Authors:

Qiuji Wu [1, 2*], Ziyue Xiang[1, 2*], Yibo Ying[1, 2*], Zhiyang Huang[1, 2], Yurong Tu[1, 2], Min Chen[1, 2], Jiahui Ye[1, 2], Haicheng Dou[1], Sunren Sheng[1], Xiaoyang Li[1], Weiyang Ying[3#], Sipin Zhu[1, 2#],

Date Issued:

August 01 2021

This document certifies that the manuscript listed above was edited for proper English language, grammar, punctuation, spelling, and overall style by one or more of the highly qualified native English speaking editors at AJESCI. Neither the research content nor the authors' intentions were altered in any way during the editing process. Documents receiving this certification should be English-ready for publication; however, the author has the ability to accept or reject our suggestions and changes. If you have any questions or concerns about this edited documen ,please contact AJESCI at www.aje-cn.com

AJESCI provides a range of editing, translation and manuscript services for researchers and publishers around the world. Our top-quality PhD editors are all native English speakers from America's top universities. Our editors come from nearly every research field and possess the highest qualifications to edit research manuscripts written by non-native English speakers For more information about our company,services and partner discounts please visit www.aje-cn.com.
